# An atypical strictosidine synthase, OsSTRL2, plays key roles in anther development and pollen wall formation in rice

**DOI:** 10.1038/s41598-017-07064-4

**Published:** 2017-07-31

**Authors:** Ting Zou, Shuangcheng Li, Mingxing Liu, Tao Wang, Qiao Xiao, Dan Chen, Qiao Li, Yanling Liang, Jun Zhu, Yueyang Liang, Qiming Deng, Shiquan Wang, Aiping Zheng, Lingxia Wang, Ping Li

**Affiliations:** 1 State Key Laboratory of Hybrid Rice, Sichuan Agricultural University, Chengdu, 611130 China; 2 Rice Research Institute, Sichuan Agricultural University, Chengdu, 611130 China; 3 Key Laboratory of Crop Genetic Resources and Improvement, Sichuan Agricultural University, Ministry of Education, Ya’an, 625014 China

## Abstract

Strictosidine synthase (STR) plays an important role in the biosynthesis of terpenoid indole alkaloids (TIAs) and is expressed in a range of active meristematic tissues of higher plants. STR proteins are involved in different physiological and biochemical pathways. However, the function of STR proteins in rice development remains poorly understood. In this study, we identified 21 possible STR-like (*OsSTRL*) family members in rice genome and found that only one gene, *OsSTRL2*, exhibited a pre-emergency specific florescence expression pattern. Tissue-specific expression profile analysis, β-glucuronidase histochemical (GUS) staining and RNA *in situ* hybridization confirmed that *OsSTRL2* was highly expressed in tapetal cells and microspores. Comparative protein sequence analysis indicated that OsSTRL2 lacked the key catalytic residue found in a typical STR (STR1), although it possessed conserved β-propellers and α-helices formed the basic structure of STR1. *OsSTRL2* knockout mutant resulted to male sterility because of the defects in anther development and pollen wall formation. Subcellular localization of OsSTRL2-YFP revealed that the OsSTRL2 protein was primarily localized in the endoplasmic reticulum (ER). Therefore, OsSTRL2 is an atypical strictosidine synthase that plays crucial roles in regulating anther development and pollen wall formation in rice.

## Introduction

Terpenoid indole alkaloids (TIAs) are among the most important secondary metabolites in plants that play important roles in the growth and reproductive development of plants^1^. Over 100 different TIAs were discovered in *Catharanthus roseus* (periwinkle)^2^. Strictosidine, identified 40 years ago as the key biosynthetic precursor of TIA, is presented in a wide variety of higher plants^1, 3^. This important molecule is generated by strictosidine synthase (STR) from tryptamine and secologanin. *STR1* (GenBank accession no. P68175) is the first STR gene isolated from *Rauvolfia Serpentine*. STR1 catalyzes the Pictet-Spengler reaction between tryptamine and secologanin and is a key enzyme for the biosynthesis of alkaloids^4–6^. Recent structural characterizations reveal that STR1 is a six-bladed four-stranded β-propeller fold from the plant kingdom, and Glu-309 is the key catalytic residue in the catalysis of STR1^4, 7^. Fifteen and six members of the STR-like family were identified in *Arabidopsis* and periwinkle, respectively^8, 9^. Among these 15 STR-like genes in *Arabidopsis*, four members exhibit relatively independent regulating patterns, play specific roles in the plant defense mechanism, and show higher similarity with those in *Drosophila melanogaster* and *Caenorhabditis elegans* than the other members^10^. STR-like members can be divided into several categories based on their different protein sequence characteristics. STR-like genes might have a variety of functions and are involved in several biochemical processes *in vivo* ^8, 11^. Hence, the unknown function of STR protein and its biochemical pathway should be determined by studying the isolation and expression of STR-like family members in different species.

*Oryza sativa* (rice) is one of the most important food crops worldwide and is an ideal model plant for monocot species^12^. Rice is also an excellent species for functional genomics in research because of the relatively small genome size of only 400 Mb^13^. Male fertility is crucial for rice, because it directly affects the rice yield. Male reproductive development is one of the most complex biological processes in higher plants that requires the participation of a large number of different genes. Male reproductive development can generally be divided into two stages. The first stage is the development of anther, which includes microspore mother cell (MMC) formation and microspore undergoing meiosis to form tetrads of microspores. The second stage is the formation of pollen, which includes the release of tetrads, microspore mitosis, anther tissue degeneration, anther dehiscence, and the release of mature pollen grains^14^. A large number of genes expressed in pre-emergency inflorescence or young flower throughout the anther development process. More than 3,000 of these genes are specifically expressed in anther before pollen maturity, whereas several of these are critical for anther and pollen development^15^. Any loss-function of these critical genes will lead to male sterility^16^.

With the completion of rice genome sequencing, a number of anther-specific genes were cloned in rice and mutated to obtain sterile male genes, such as *UDT1*, *TDR*, *EAT1*, *TIP2*, *PTC1*, *CYP703A3*, *CYP704B2*, *DPW*, and *OsABCG15* ^17–27^. *UDT1* is expressed in early meiosis and plays an important role in the development of tapetum^18^. Three basic helix-loop-helix family genes, *TDR*, *EAT1*, and *TIP2*, play central roles in controlling tapetum programmed cell death (PCD). Loss-function of each genes delays the degradation of the tapetum and results in pollen abortion. *TIP2* is expressed at the upstream of *TDR* and *EAT1*, and can interact with *TDR* to form a heterodimer, and thus activate *EAT1* transcription by binding to its promoter^17, 19, 26, 27^. *PTC1* encodes a PHD-finger protein, which is specifically expressed in tapetal cells and microspores during anther development, and controls the programmed tapetal development and functional pollen formation^20^. Two cytochromes, *CYP703A3* and *CYP704B2*, play a similar role in the formation of both cuticle and exine during plant male reproductive development^21, 22^. *DPW*, a novel fatty acid reductase, is important for primary fatty alcohol synthesis for anther cuticle and pollen sporopollenin biosynthesis^23, 24^. *OsABCG15* functions as the ATP-binding cassette (ABC) transporter of lipid precursors in the formation of anther cuticle and pollen exine, and it is preferentially expressed in the tapetum of young anther^25^.

A genome-wide analysis of the rice STR-like family has not yet been reported, although the sequencing of the rice genome was completed in 10 years ago^13^. In the present study, we performed the first genome-wide analysis of the rice STR-like family (*OsSTRL*) and identified 21 possible family members in the rice genome. We also present evidence that *OsSTRL2* plays important roles in anther development and pollen wall formation. Among the 21 *OsSTRL* members, only *OsSTRL2* showed a specific and strong transcript signal for pre-emergency inflorescence, young panicles, and anthers. Although OsSTRL2 contains the conserved six repetitive β-propellers and three α-helices that formed the basic structure of STR1, this protein lacks the key catalytic residue found in STR1. The knockout of *OsSTRL2* caused male sterility due to the defects of the anther wall and pollen exine development. GUS-reporter gene driven by its native promoter and RNA *in situ* hybridization indicated that *OsSTRL2* was specifically expressed in tapetal cells and microspores. Moreover, OsSTRL2 was predominantly targeted to the ER. Overall, our results suggest that OsSTRL2 is an atypical strictosidine synthase that is essential for anther development and pollen wall formation in rice; however, this protein might not act similar to a typical strictosidine synthase in its biochemical pathways.

## Results

### Genome-wide identification of 21 *OsSTRL* genes in rice

BLASTP analysis was performed with STR1 protein sequence to identify the *OsSTRL* members in rice. Twenty-one putative OsSTRL protein sequences were obtained (Table 1) from the MSU Rice Genome Annotation Project Database (RGAP; http://rice.plantbiology.msu.edu/)^28^. To further confirm the reliability of these candidates, we performed conserved domain analysis by using the Simple Modular Architecture Research Tool (SMART)^29^. All OsSTRLs were detected within the “Str_synth” domain (PF03088.9) in their protein sequences. Through genomic distribution analysis, we found that the 21 *OsSTRL* members were distributed across chromosomes 1, 3, 6, 7, 8, 9, 10, 11, and 12. These genes were not found in the other three chromosomes (Table 1).

**Table 1:** The rice OsSTRL genes information.

Serial no.	Generic name	Gene ID in RGAP	RGAP CDS Coordinates (5′ to 3′)	Chromosamal location	Proposed clade or singleton/group
1	OsSTRL1	LOC_Os01g50330	28892212–28891193	1	Clade II/group I
2	OsSTRL2	LOC_Os03g15710	8666146–8664064	3	Clade III/group I
3	OsSTRL3	LOC_Os03g53950	30929823–30927223	3	Singleton/group I
4	OsSTRL4	LOC_Os06g35950	20983248–20982674	6	Group II
5	OsSTRL5	LOC_Os06g41820	25081930–25080893	6	Clade I/group I
6	OsSTRL6	LOC_Os06g41830	25085537–25086526	6	Clade I/group I
7	OsSTRL7	LOC_Os06g41850	25093242–25094294	6	Clade I/group I
8	OsSTRL8	LOC_Os07g35970	21519953–21518519	7	Clade I/group I
9	OsSTRL9	LOC_Os07g35990	21532128–21531376	7	Clade I/group I
10	OsSTRL10	LOC_Os07g36040	21558783–21559529	7	Clade I/group I
11	OsSTRL11	LOC_Os07g36060	21564056–21565111	7	Clade I/group I
12	OsSTRL12	LOC_Os07g42250	25287511–25290131	7	Clade III/group I
13	OsSTRL13	LOC_Os08g07810	4371022–4372930	8	Clade II/group I
14	OsSTRL14	LOC_Os08g34330	21551327–21549576	8	Clade II/group I
15	OsSTRL15	LOC_Os09g20684	12454365–12460996	9	Clade II/group I
16	OsSTRL16	LOC_Os09g20700	12466523–12467998	9	Group II
17	OsSTRL17	LOC_Os09g20720	12484352–12484831	9	Clade II/group I
18	OsSTRL18	LOC_Os09g20810	12536621–12539847	9	Singleton/group I
19	OsSTRL19	LOC_Os10g39710	21224367–21226941	10	Singleton/group I
20	OsSTRL20	LOC_Os11g04660	1986480–1988225	11	Clade IV/group I
21	OsSTRL21	LOC_Os12g04424	1882578–1888289	12	Clade IV/group I

The number designation was based on the position from the top to the bottom on the rice chromosomes 1 to 12.

To understand the relationship among the *OsSTRL* genes, we used corresponding protein sequences from these genes and STR1 to perform peptide alignment by employing Clustal W (www.ebi.ac.uk/Clustalw). The result showed significant conservation within the strictosidine synthase domain among the OsSTRL proteins (Supplementary Figure﻿ 1). A neighbor-joining (NJ) phylogenetic tree was constructed by using the above multiple sequence alignment results with bootstrap analysis (1,000 replicates) (Supplementary Figure 2) to obtain clues about the evolutionary history of the *OsSTRL* genes. To analyze the conserved motifs of OsSTRL proteins, we employed the MEME motif search tool to investigate the shared motifs^30^. The *OsSTRL* genes were manually divided into two major groups based on the bootstrapping values of phylogram (Supplementary Figure 2) and the conserved motif distribution of OsSTRL proteins (Supplementary Figure 2). Group I is composed of four clades and three singletons, namely, *OsSTRL19*, *OsSTRL3*, and *OsSTRL18* (Table 1 and Supplementary Figure 2); STR1 acted as a singleton in this group (Table 1 and Supplementary Figure 2), which is probably due to the divergence of ancient evolution between rice and *Rauvolfia serpentine*. By using the MEME motif search tool, we identified that: (1) motif 3 was conserved in all the members of groups I and II; and (2) motifs 2 and 3 comprised the strictosidine synthase domain in STR1 and most of OsSTRL proteins (Supplementary Figure 2).

### OsSTRL2 is an atypical strictosidine synthase that is specifically expressed in the developing anther in rice

**Figure 1:**
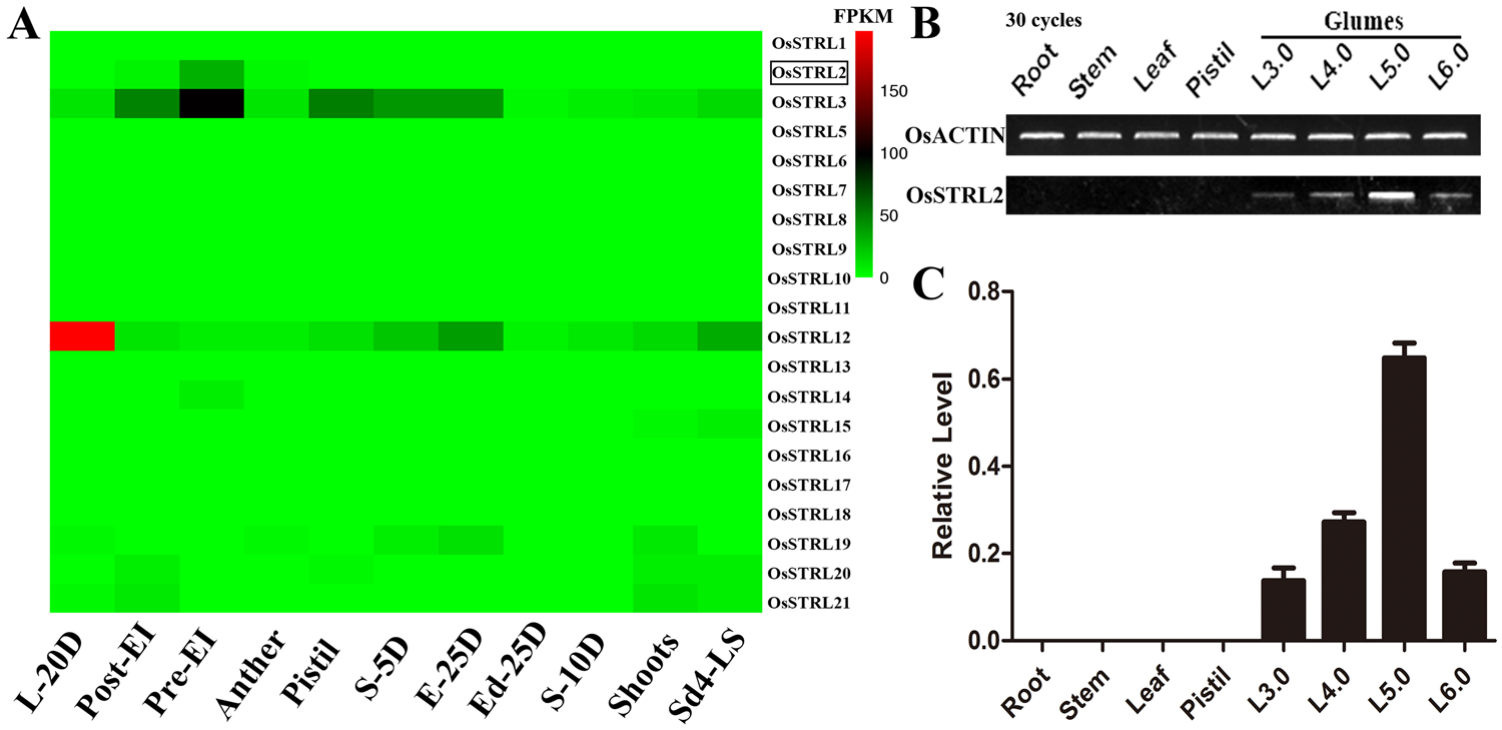
Expression patterns of *OsSTRL* genes and expression analysis of *OsSTRL2*. (**A**) Heat map of *OsSTRL* members’ expression patterns. The absolute FPKM of each OsSTRL gene from NGSTD were used for the heat map analyses. The scale indicates the FPKM expression values. L-20D, leaves at 20 days; Post-EI, post-emergency inflorescence; Pre-EI, pre-emergency inflorescence; S-5D, seed at 5 days after pollination; E-25D, Embryo at 25 days after pollination; Ed-25D, endosperm at 25 days after pollination; S-10D, seed at 10 days after pollination; Sd4-LS, seedling at the 4-leaf stage. (**B** and **C**) Semi RT-PCR and qPCR for spatial and temporal expression analysis of OsSTRL2. L3.0, glume length of 3.0 mm; L4.0, glume length of 4.0 mm; L5.0, glume length of 5.0 mm; L6.0, glume length of 6.0 mm. The cycles of semi RT-PCR are 30.

Given that protein functions are often correlated with the gene expression patterns, we performed digital expression analysis of *OsSTRL* members by using the RNAseq data from the Next Generation Sequencing Transcriptome Data in the RGAP (NGSTD, (http://rice.plantbiology.msu.edu/expression.shtml). *OsSTRL* transcripts obtained from NGSTD (Fig. 1A) suggested that two members (*OsSTRL3* and *OsSTRL12*) had ubiquitously strong expressions, whereas the genes in clades I and II (Table 1 and Supplementary Figure 2) had relatively low expressions in the studied tissues. We manually excluded *OsSTRL4* from the heat map in Fig. 1A because this gene had 0 FPKM expression values in all tissues in NGSTD. Further investigation of the expression profile of *OsSTRL* members through semi-quantitative real time PCR (semi-RT-PCR, Supplementary Figure 3) showed that *OsSTRL19*, *OsSTRL20*, and *OsSTRL21* were expressed in all the tissues investigated. *OsSTRL5* and *OsSTRL6* showed a similar expression pattern of having a specific but weak transcription signal in the young panicles. The expression values of *OsSTRL8* and *OsSTRL11* were extremely low in all tissues, although they were expressed in the anthers. *OsSTRL14* and *OsSTRL15* were expressed in young panicles and in other vegetative organs (leaf, stem, and root). Interestingly, only *OsSTRL2* showed a markedly specific transcript signal in both pre-emergency inflorescence, young panicles, and anthers. For further validation, we examined *OsSTRL2* expression patterns by using semi-RT-PCR (Fig. 1B) and quantitative real time PCR (q-PCR) (Fig. 1C) in a range of rice organs, including vegetative tissues, namely, roots, stems, and leaves, and reproductive tissues, such as pistils and anthers, at different stages of development. The results showed an exclusive and obvious expression level of *OsSTRL2* in the anther with glume lengths of 3.0 mm to 6.0 mm, indicating that *OsSTRL2* might have a function in anther development.

**Figure 2:**
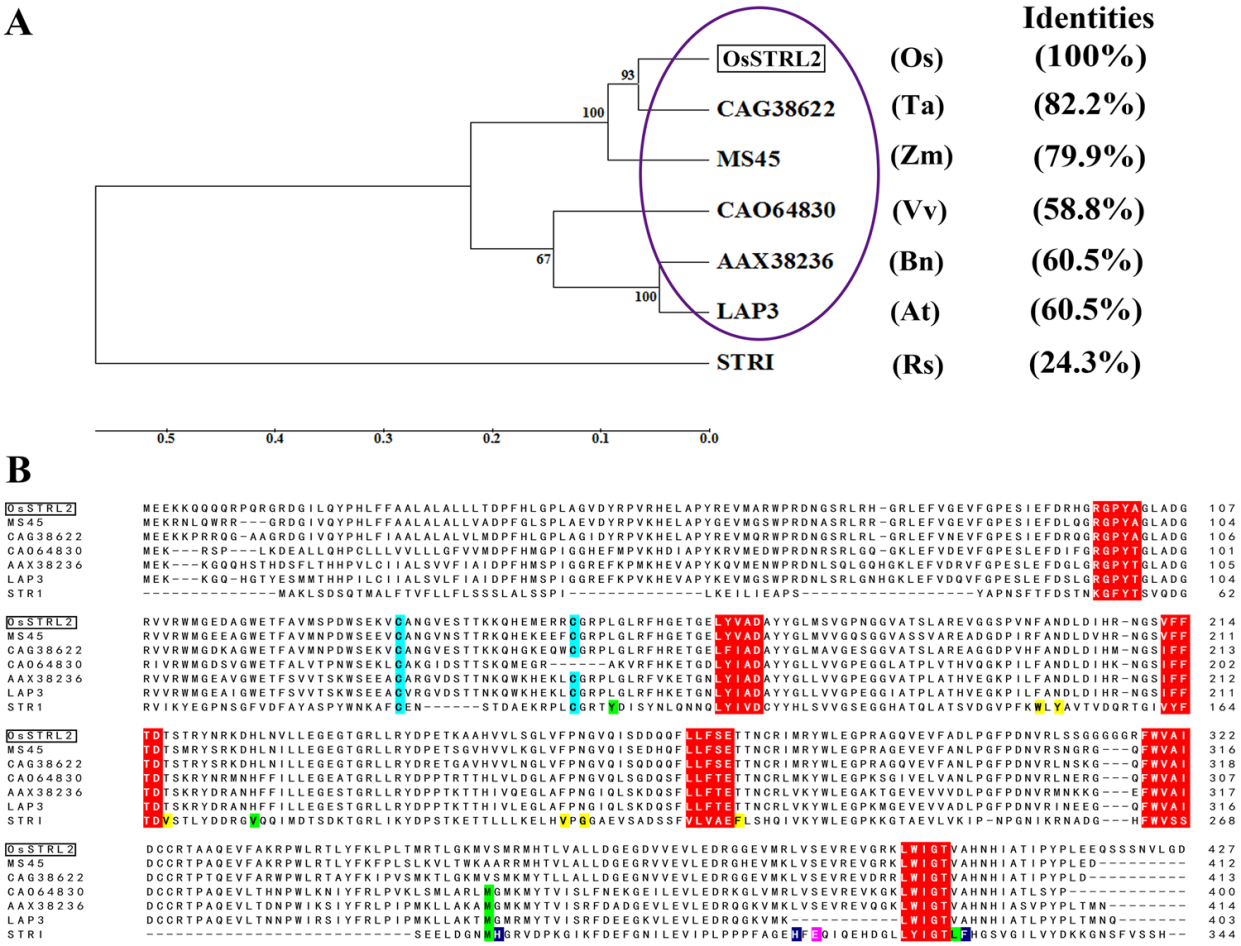
Phylogenetic analysis and alignment of OsSTRL2-related proteins with STR1. (**A**) MEGA5 program, which used the Neighbor–Joining method with default parameters in addition to 1,000 bootstrap replications to generate the phylogenetic tree of OsSTRL2, OsSTRL2-homologies in different species and STR1. The purple ellipse indicates the genes in the same branch. The numbers at the nodes indicate the bootstrap value. The percentage identities represent the similarities between corresponding protein sequences and OsSTRL2. At, *Arabidopsis thaliana*; Bn, *Brassica napus*; Os, *Oryza sativa*; Rs, *Rauvolfia serpentine*; Ta, *Triticum aestivum*; Vv, *Vitis vinifera*; Zm, *Zea mays*. (**B**) Alignment of seven proteins in Fig. 2A using Clustalw2. Red background characters indicate the motifs for β-propeller folds. Light blue background characters indicate the conserved residues forming the disulfide bridge that pulls two α-helices together. Green background characters indicate the residues’ contact with the terpenoid part of secologanin. Yellow background characters indicate the residues’ contact with the indole part of strictosidine. Deep blue background characters indicate the residues’ contact with the glucose moiety. Magenta background character indicates the key catalytic residue of typical strictosidine synthase.

Using the full-length amino acids of OsSTRL2 as a query, we performed a BLASTP search according to the Phytozome database (www.phytozome.net) to explore the potential function of *OsSTRL2*. Five putative homologs of OsSTRL2 were obtained in *Vitis vinifera* (Vv), *Zea mays* (Zm), *Arabidopsis thaliana* (At), *Triticum aestivum* (Ta), and *Brassica napus* (Bn). The homolog amino acid sequences, including STR1 and OsSTRL2, were subsequently aligned with Clustalw2^31^, and a phylogenetic tree was generated from the MEGA5^32^. The phylogenetic tree showed that OsSTRL2 and its related proteins, which were identified by Phytozome BLASTP, were grouped into the same clade, whereas only STR1 belonged to the other branch (Fig. 2A). OsSTRL2 shared ~82.2%, ~79.9%, ~58.8%, ~60.5%, and ~60.5% identities with the OsSTRL2-related protein sequences in Ta, Zm, Vv, Bn, and *Arabidopsis*, respectively (Fig. 2). However, only 24.3% identity was found between OsSTRL2 and STR1 proteins (Fig. 2). The previous 3D-structural and functional analyses suggested that the six-bladed β-propeller and three α-helices formed the basic structure of the STR1 protein; some important residues were located in the STR1 substrate-binding region, in which the Glu-309 was experimentally proven to be the key catalytic residue^4^. Although the motifs for β-propeller folds and the residues forming a disulfide bridge that pulls two α-helices together were conserved^4–7^, several variations were found within the substrate-binding region in both OsSTRL2 and its homologues of the same branch, including the key site of Glu-309 (Fig. 2B). All OsSTRL members lack the residue Glu-309 which acts as the key catalytic residue of STR1 protein^4^ (Supplementary Figure 1). Moreover, the critical residues that were not found in the OsSTRL2 protein were also absent in the two *Arabidopsis* strictosidine synthase-like (ATSsl) proteins, ATSsl7 and ATSsl14 (Supplementary Figure 4), which do not exhibit STR enzymatic activity^33^. These results suggested that *OsSTRL2*, which was specifically expressed in the developing anther, might be an atypical strictosidine synthase.

### Knockout of OsSTRL2 in rice causes male sterility

**Figure 3:**
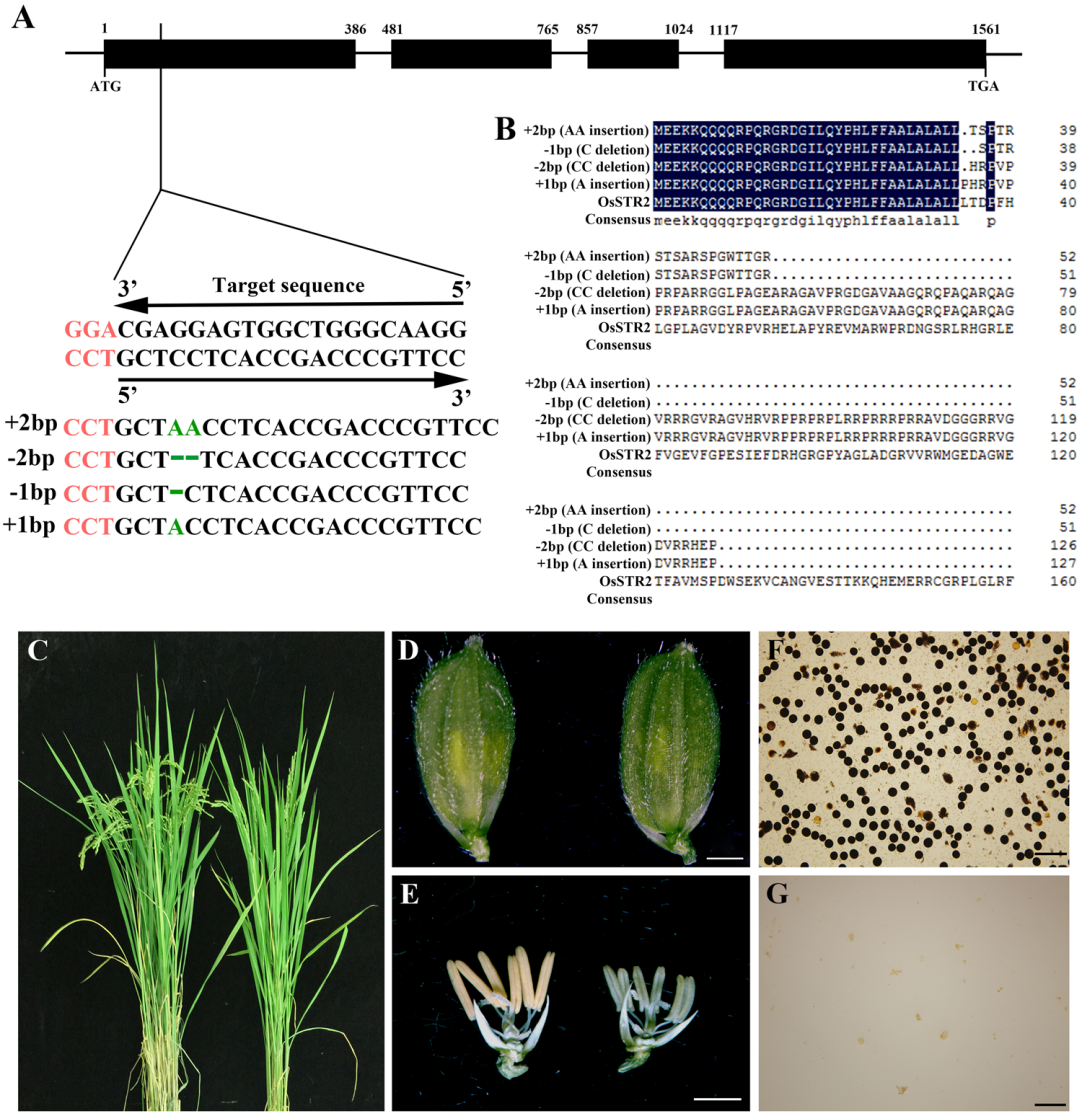
Sequence analysis and phenotypic observation of *OsSTRL2* CRISPR/Cas9-mediated mutant. (**A**) Gene structure of *OsSTRL2* and mutation analysis of *OsSTRL2* gene in transgenic plants. The sequence (5ʹ-CCTGCTCCTCACCGACCCGTTCC-3ʹ) located in the first exon of the OsSTRL2 gene was selected as the target site of sgRNA. The black boxes indicate the exons. The red characters indicate the PAM. The green characters indicate the four different types of mutation events generated by CRISPR-Cas9 in the mutants (The detailed information of four different mutation types are listed in Supplementary table S2). (**B**) Putative amino acid sequences alignment of four types of mutation as described in Fig. 3A. The sequences were aligned using DNA-MAN and displayed using BOXSHADE. The deep blue boxes indicate the 100% identities of amino acids. (**C**) Phenotypic comparison of the WT (left) and the mutant plant (right) at the maturity stage. (**D**) Panicle comparison of the WT (left) and the mutant (right) at the heading stage. (**E**) Floral comparison of the WT (left) and the mutant (right) (with the palea and lemma removed) at the heading stage. (**F** and **G**) I_2_–KI pollen staining for the WT and the mutant. Bars = 0.20 mm (**D** and **E**); 30 μm (**F** and **G**).

The loss-of-function mutations in At *Lap3* and Zm *Ms45*, two putative orthologues of OsSTRL2 (Fig. 2A), resulted in male sterility and defects in the pollen wall formation^34, 35^, suggesting that *OsSTRL2* might share conserved protein functions with *LAP3* and *MS45*. To investigate the role of *OsSTRL2* in rice development, we obtained 29 independent *OsSTRL2* knockout plants by using the CRISPR/Cas9-mediated genome-editing tool. The target site in the leaves of T_0_ transgenic lines were sequenced to determine the mutation of the target sequence (Fig. 3A). Mutations in the target site were detected in 22 out of 29 T_0_ plants – four plants carried homozygous mutations, 15 plants carried bialleic mutations, and three plants carried heterozygous mutations (Supplementary Table 1). Among these mutants, four different mutation types were identified in the target site (Fig. 3A and Supplementary Table 2), including two adenosine insertions, two cytosine deletions, one cytosine deletion, and one adenosine insertion. These mutations yielded premature stop codons and predictions to produce four types of truncated polypeptides (Fig. 3B and Supplementary Table 2).

No difference was observed between the wild-type plants *Oryza sativa japonica* cultivated variety Nipponbare (WT) and the T_0_ plants during vegetative growth (Figs. 3C and D). However, all T_0_ plants carrying homozygous or biallelic mutations had short whitish anthers with very few pollen grains, which could not be stained by the I_2_/KI solution (Figs. 3E–G). By contrast, all heterozygous T_0_ plants exhibited normal fertility as observed in the WT (Supplementary Table 1). Similar results were observed in several F2 populations generated by the crosses of these mutants and the WT, thereby further confirming the association of phenotype and genotype. The findings are as follows: (1) the binary construct for targeting the *OsSTRL2* site had a high targeted editing efficiency, (2) the phenotype of male sterility in the knockout plants was caused by the mutations revealed in the gene *OsSTRL2*, and (3) *OsSTRL2* played an essential role in controlling male fertility.

### Defects of anther wall and pollen exine development in *OsSTRL2* knockout lines

**Figure 4:**
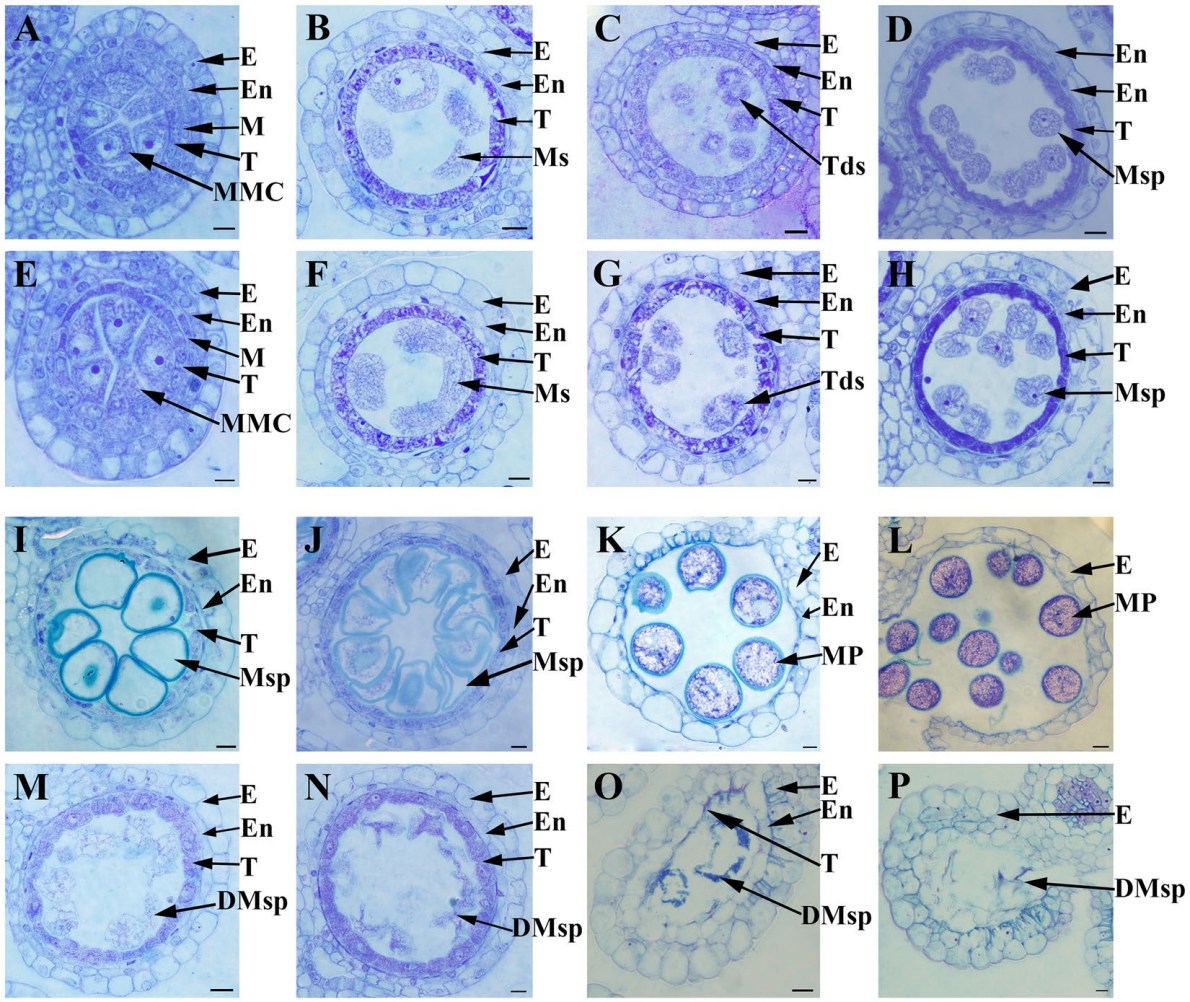
Semi-thin section comparison of anther development between the WT and *OsSTRL2* knockout mutant. Comparison of the eight stages of anther development between the WT and the mutant. (**A** and **E**) stage 7; (**B** and **F**) stage 8a; (**C** and **G**) stage 8b; (**D** and **H**) stage 9; (**I** and **M**) stage 10; (**J** and **N**) stage 11; (**K** and **O**) stage 12; and (**L** and **P**) stage 13. The WT sections are shown in (**A**–**D** and **I**–**L**), and the sections of the mutant are shown in (**E**–**H** and **M**–**P**). DMsp, degenerated microspores; E, epidermis; En, endothecium; M, middle layer; MMC, microspore mother cell; MP, mature pollen; Ms, microsporocyte; Msp, microspore; T, tapetum; Tds, tetrads. Bars = 20 μm.

We performed transverse section analysis for the anthers of the WT and the mutant to investigate the defects of pollen development in the knockout mutant of *OsSTRL2*. The previous study classified the rice pollen and anther development into 14 stages from the formation of stamen primordium to the release of mature pollen during anther dehiscence^36, 37^. At stage 7, meiosis was initiated by the WT MMC within the locule that was surrounded by the four-layered anther wall of epidermis, endothecium, middle layer, and tapetum from the surface to the interior (Fig. 4A). MMC subsequently formed dyads and tetrads after two steps of meiosis (Figs. 4B and C). Towards the end of stage 8b, the tapetal cells initiated linearization and centralization, and tapetum had close cell arrangement^36, 37^ (Fig. 4C).

No detectable defects were observed between the WT and the mutant anthers until the anther stage 9 (Figs. 4A–C and E–G). However, the mutant anther showed clear morphological differences after stage 8b. At stage 9, the mutant microspores had a slightly wrinkled shape, although these were released normally as with WT (Figs. 4D and H). In stage 10, the WT microspores were vacuolated and became round-shaped. The tapetal cells started to deteriorate (Fig. 4I), and asymmetric mitosis occurred in vacuolated microspores, which formed generative and vegetative cells at the stage 11 (Fig. 4J). The tapetum of WT gradually thinned from stages 10 to 11(Fig. 4I–J). By contrast, the wrinkled microspores in the mutant had more observable shrinkage deformity from stages 10 to 11 (Figs. 4M,N). The delayed degradation of the tapetum was also observed in the mutant at the stage 10. In contrast to that in the WT, the mutant anthers still maintained a thick layer of tapetal cells in stage 11 (Figs. 4M,N). At stage 12, the microspores of the WT were round, enlarged, enriched in starch, and developed into mature pollens that filled the anther locule (Fig. 4K). However, the microspores of the mutant were extremely wrinkled, and the inner surface of mutant anther wall still had an incompletely degraded tapetum (Fig. 4O). At stage 13, the defected microspores of the mutant wrinkled into a distorted and vermiform shape, which thereby emptied the locules of the mutant anthers, whereas the WT anthers were filled with mature pollen and started dehiscence (Figs. 4L and P).

**Figure 5:**
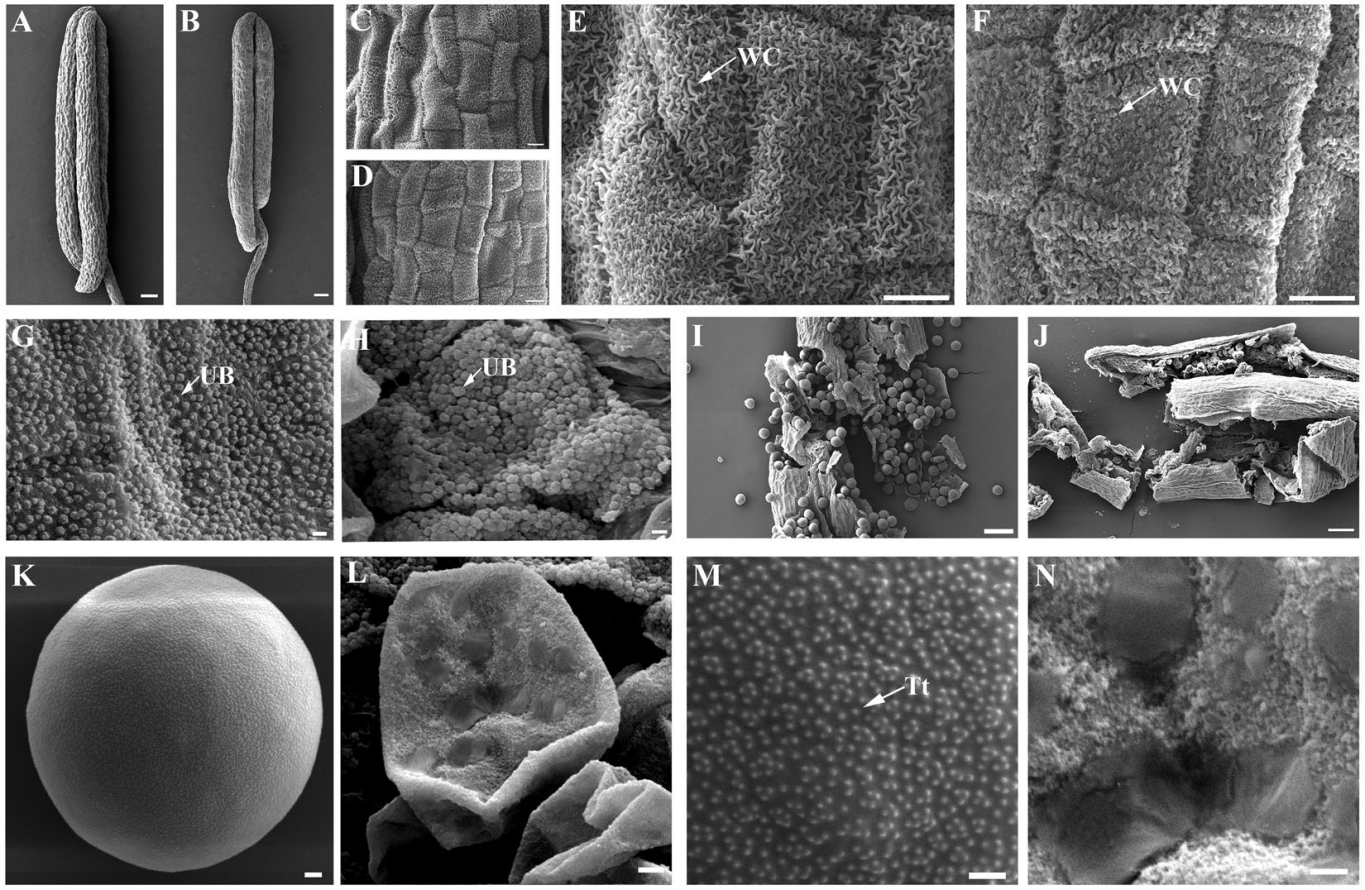
SEM observation for the WT and *OsSTRL2* knockout mutant anther and pollen at the stage 12. (**A** and **B**) Anthers of the WT and mutant. (**C** and **D**) Anther epidermis of the WT and mutant. (**E** and **F**) In the enlarged images of epidermal surface of the WT and mutant anthers, the arrows indicate the wax crystal (WC). (**G** and **H**) In the inner surface of the WT and mutant anthers, the arrows indicate the ubisch body (UB). (**I** and **K**) Pollen grains in the WT anthers. (**J** and **L**) Pollen grains in the mutant anthers. (**M** and **N**) Outer surface of pollen grains of the WT and mutant; the arrows indicate the tectum (Tt). Bars = 100 μm (**A**, **B**, **I** and **J**); 10 μm (**C**–**F**); 2 μm (**K** and **L**); 1 μm (**G**, **H**, **M**, and **N**).

Scanning electron microscopy (SEM) was used during stage 12 anther samples of the WT and the mutant to obtain detailed information on the defects of the anther wall and pollen exine in the mutant. In agreement with the phenotypic observation results (Fig. 3E), the anthers of the mutant (Fig. 5B) were also shorter and smaller than those in the WT (Fig. 5A). In contrast to those with the WT (Figs. 5C and E), the wax crystals of the mutant anthers exo-surface were crowded and less regularly arranged, leading to a compact cell arrangement of anther epidermis (Figs. 5D and F). Orbicules are critical for exporting materials from tapetum to microspores^38^. However, compared with the smooth and regularly arranged orbicules emerging in the WT (Fig. 5G), slightly rough and congested orbicules were formed by the mutant inner locule side of tapetum surface (Fig. 5H). Consistent with the transverse section results (Fig. 4P), the mutant produced less, deflated, and adherent pollen grains (Fig. 5J), whereas the pollen grains in the WT were abundant and round-shaped (Fig. 5I). The exine, mainly composed of sporopollenin, is the most important outer layer of the pollen wall^39, 40^. We scanned the surfaces of pollen grains in the WT and the mutant. The pollen grains in the WT showed elaborate exine patterning and roof-like tectum structure on their surface (Figs. 5K and M). By contrast, the exine surface was discontinuous and plush-like, and the roof-like tectum structure was generally lost in the mutant (Figs. 5L and N). These results suggested that OsSTRL2 was involved and played an important role in the network of regulating anther wall formation, tapetum degeneration and pollen exine development in rice.

### *OsSTRL2* was specifically expressed in tapetum and microspores

**Figure 6:**
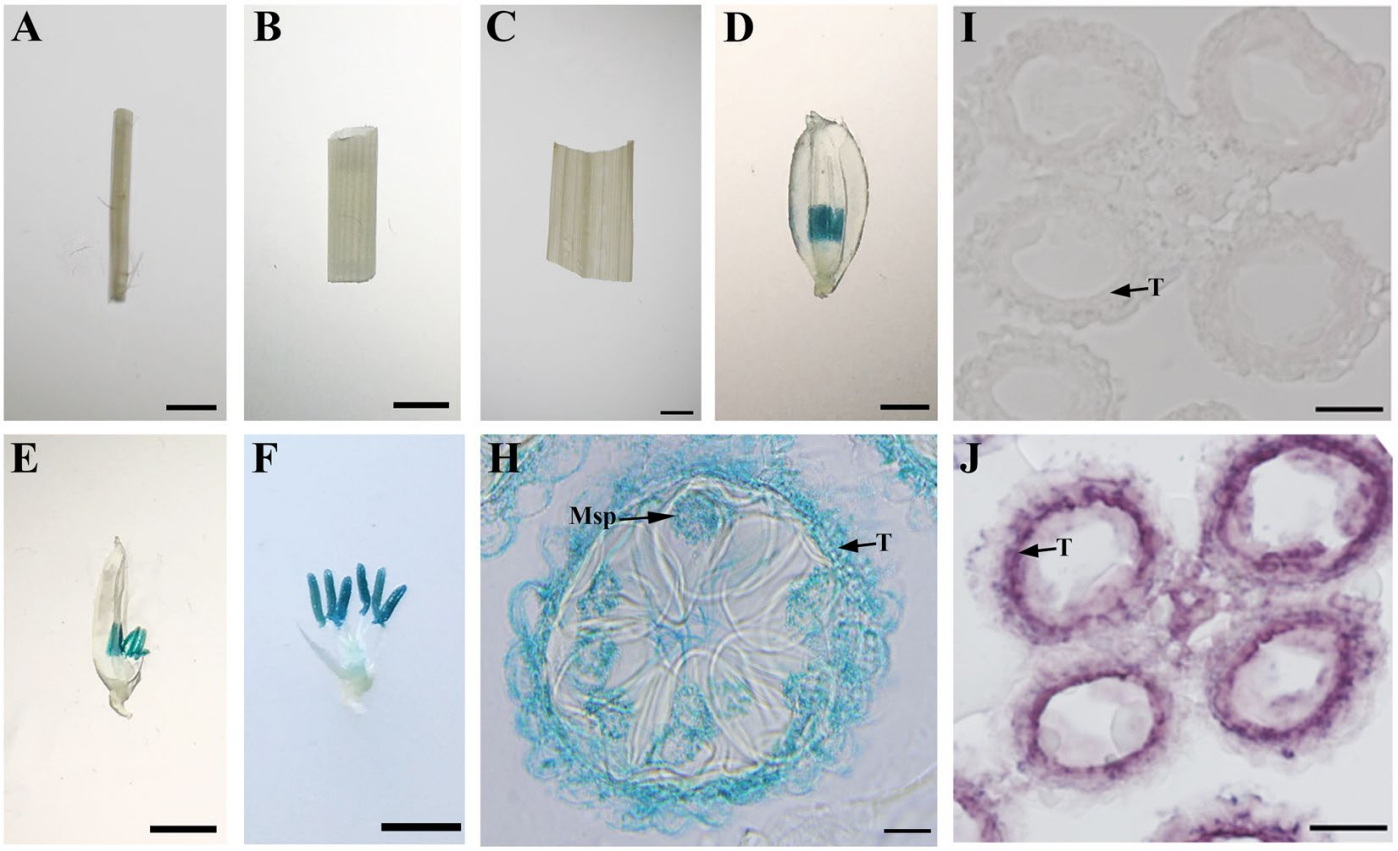
GUS-reporter analysis and RNA *in situ* hybridization of *OsSTRL2*. (**A**–**H**) GUS expression (blue stained) patterns of root, stem, leaf, floret, spikelet (with the palea removed), spikelet (with the palea and lemma removed) and the section of GUS-staining anther of the OsSTRL2pro::GUS transgenic line, respectively. RNA *in situ* hybridization using the WT anther sections. (**I**) Sense probe. (**J**) Antisense probe. Bars = 3 mm (**A**–**F**); 30 μm (**H**); 150 μm (**I** and **J**).

To directly determine the spatial expression pattern of *OsSTRL2*, we applied promoter-GUS reporter system to detect its transcriptional activity in the tissues examined with semi-RT-PCR and q-PCR analyses. The transgenic lines containing a GUS-reporter gene driven by the *OsSTRL2* promoter further confirmed the specific expression of *OsSTRL2* in the developing anther (Figs. 6A–F). Particularly, the section of the GUS-staining anther showed that *OsSTRL2* was expressed in the tapetum and microspores (Fig. 6H). RNA *in situ* hybridization was performed using the WT anther sections to precisely investigate the spatial and temporal expression patterns of *OsSTRL2*. The results revealed that *OsSTRL2* expression was strong and specifically occurred in the tapetum at stage 8 (Fig. 6J). All these results support the functional role of *OsSTRL2* in regulating pollen development.

### OsSTRL2 protein is primarily localized to the ER

**Figure 7:**
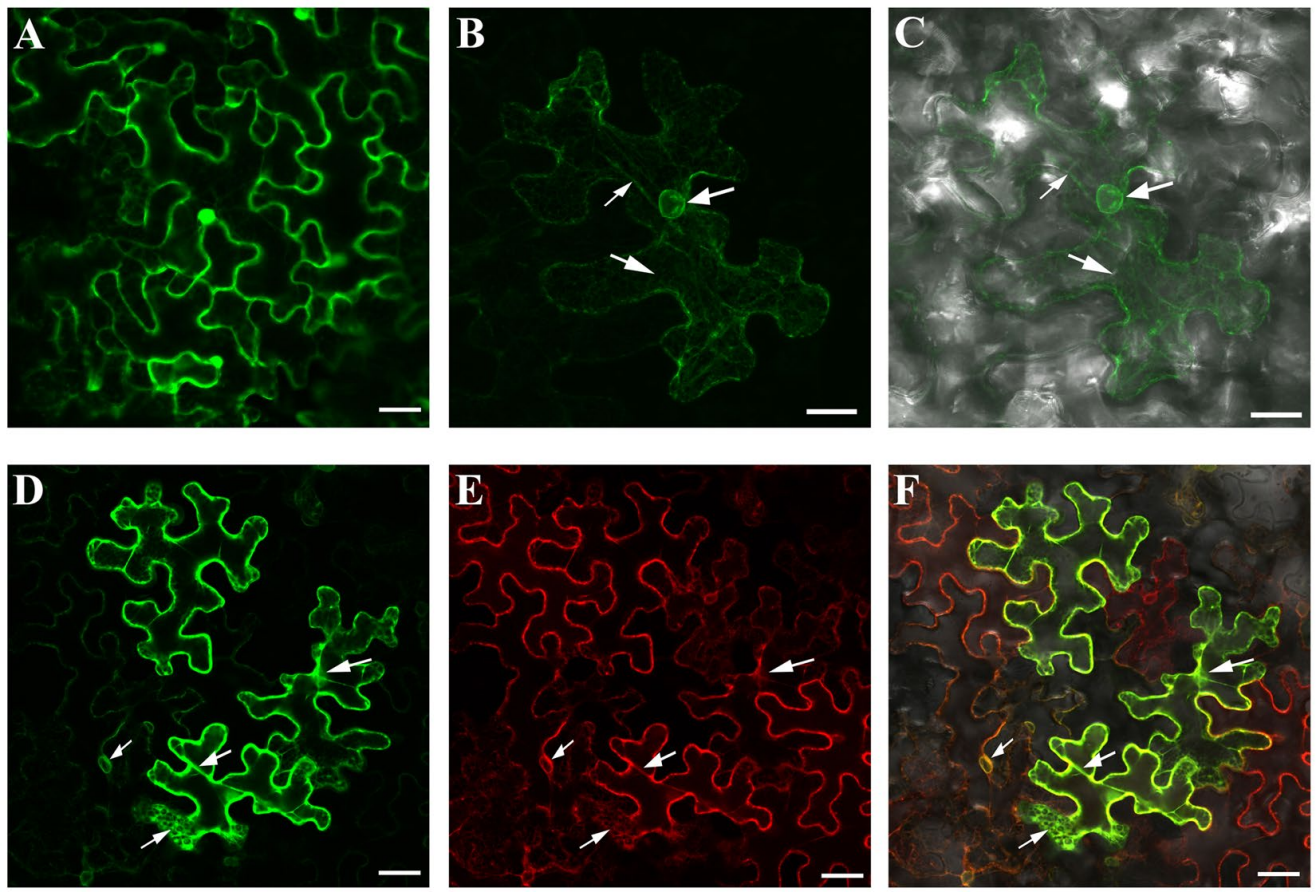
Subcellular localization of OsSTRL2-YFP in tobacco leaf epidermal cells. Confocal images of tobacco leaf epidermis cells after 72 hours of infection were shown. (**A**) YFP signal of 2 × 35 S::YFP as control. (**B**) YFP signal of 2 × 35 S::OsSTRL2-YFP. (**C**) Merged image of (**B**) and bright field. (**D** and **E**) The YFP and RFP signal of co-expressed 2 × 35 S::OsSTRL2-YFP and ER-marker. (**F**) Merged signal of (**D** and **E**) and bright field. Bars = 10 μm (**A**, **D**, **E** and **F**); 25 μm (**B** and **C**).

To determine the subcellular localization of OsSTRL2, we generated a yellow fluorescent protein (YFP) fused to the C-terminal of OsSTRL2, under the control of the cauliflower mosaic virus double 35 S promoter (2 × 35S::OsSTRL2-YFP). Transiently expressed results showed that the YFP signal was localized at the endoplasmic reticulum (ER) that surrounds the nuclei (ER-ring) in the tobacco leaf epidermal cells (Figs. 7B and C) and 2 × 35S:: YFP as the control (Fig. 7A). Moreover, YFP signals were also detected on some tubular and cisternal patterns similar to the ER-like structure (Figs. 7B and C). The co-infiltration of Agrobacterium containing the ER-marker and 2 × 35S::OsSTRL2-YFP to the epidermal cell layers of tobacco leaves was conducted to confirm the complex localization of OsSTRL2. Confocal laser-scanning microscopy indicated that the YFP signals (Fig. 7D) were well merged with the ER red fluorescent protein (RFP) signals (Figs. 7E and F). Thus, OsSTRL2 proteins were primarily localized in the ER-ring and in the tubular and cisternal ER structures. These results also suggested that the ER might be an important site for the regulation of pollen exine formation.

## Discussion

Strictosidine is the common precursor of all indole alkaloids and is synthesized from tryptamine and secologanin under STR catalysis, which is the key step of TIA biosynthesis^1, 3^. The plant *STR* is expressed in active meristematic tissues, such as young leaves, flower buds, shoot tips, and petals during plant development, and has a close connection with cell division^41^.

The genome-wide analysis showed that rice contains at least 21 *OsSTRL* genes that were divided into two groups (Table 1 and Supplementary Figure 2). The large family members of *OsSTRL* indicated their essential role in rice growth and metabolism. The expression patterns analysis of *OsSTRL* genes from public data and semi RT-PCR (Fig. 1 and Supplementary Figure 4) showed that five *OsSTRL* members (*OsSTRL3*, *OsSTRL12*, *OsSTRL19*, *OsSTRL20*, and *OsSTRL21*) were constitutively expressed in all tissues, whereas nine members (*OsSTRL1*, *OsSTRL4*, *OsSTRL7*, *OsSTRL9*, *OsSTRL10*, *OsSTRL13*, *OsSTRL16*, *OsSTRL17*, and *OsSTRL18*) displayed extremely weak transcription signals in any of the tissues investigated, which suggests that these genes might be expressed at specific developmental stages or under special conditions. However, only *OsSTRL2* had strong and specific transcription signals in pre-emergency inflorescence, young panicles, and anther (Fig. 1A). Similar to male sterility-related genes that were recently identified, *OsSTRL2* was preferentially expressed in the developing anther before mature pollen formation as suggested by the additional investigation via semi-RT-PCR and q-PCR (Figs. 1B and C).

Protein sequence alignment analysis reveals that the OsSTRL2 possesses high identities of 79.9% and 60.5% of MS45 (maize) and LAP3 (*Arabidopsis*), respectively (Fig. 2). Both *MS45* and *LAP3*, also annotated in the STR family, are specifically expressed in their anthers, and the mutants of these two genes are male sterile^34, 35^. In addition, the knockout of *OsSTRL2* in rice causes abnormal male reproductive development phenotypes, including smaller and white anthers and few and defective pollen grains (Figs. 3G, 4P and 5J), suggesting that *OsSTRL2* plays vital roles in anther development and pollen formation.

Tapetum, the innermost cell layer of anther wall, produces sporopollenin precursors that are transported to the developing microspores for pollen exine formation^42–44^. Ubisch body, also named orbicule, is located on the inner surface of the tapetal cells and is only a few microns (μm) in size^45^. Ubisch body is one of the most essential by-products in pollen wall sporopollenin synthesis^46^. Within the *OsSTRL2* knockout mutant anther, tapetum degeneration was delayed (Figs. 4M and N), and the ubisch bodies in the mutant inner locule side of tapetal surface had irregular shape and chaotic arrangement compared with those in the WT (Fig. 5H). Moreover, epidermal waxes can primarily protect the land plants from water loss and can also be involved in plant defenses against pathogens^47, 48^. Compared with the WT anther, the crowded permutation and distorted appearance of wax crystals on the mutant outer epidermis, might be the main reason for the smaller anther exo-surface cell size in the mutant anther (Figs. 5C–F). These results indicated that *OsSTRL2* has a key function in the biological pathway of tapetum PCD and ubisch body formation. *OsSTRL2* loss-function might affect the expansion and morphology of the anther epidermal cells by producing abnormal wax crystals.

Pollen wall is a surrounded lipidic structure of male gametophyte that plays an essential role in protecting pollen from various environmental stress and bacterial attack. Pollen wall consists of two layers: the outer exine, which contains tectum and baculum; and the inner intine^39, 40, 49, 50^. In *Arabidopsis*, PKSA and PKSB, two plant type III polyketide synthases (PKSs), mediate the biochemical reactions to induce the synthesis of pollen fatty acids and phenolics found in exine^51, 52^. As an Acyl-CoA synthetase catalyzing the fatty acyl-CoA ester biosynthesis reaction in *Arabidopsis* tapetal cells, ACOS5 plays a central role in generating sporopollenin monomers that can be exported to microspores and regulate the formation of pollen exine^53, 54^ *. LAP3* is a homolog of *OsSTRL2* in *Arabidopsis* (Fig. 2). *Lap3* mutant plants produce less adherent pollen grains because of their thinner pollen exine walls^34^. Similar to that in *lap3*, the *OsSTRL2* knockout mutant pollen grains displayed structurally weakened exine that lacks tectum (Fig. 5N). This defect might cause pollen grains to rupture easily and cause adhesion (Figs. 3G, 4M–P and 5J). By contrast, the pollen grains in WT were round and arranged by normal roof-like tectum on their outer exine surface (Fig. 5M). These results suggested that *OsSTRL2* might use similar regulatory pathways for pollen exine formation in *Arabidopsis*.

**Figure 8:**
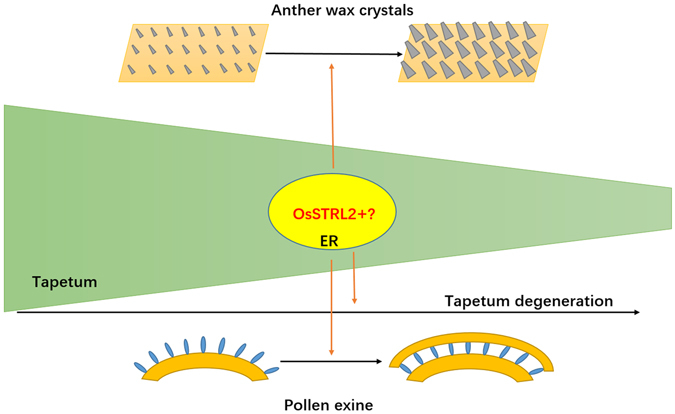
Model for the role of OsSTRL2 during tapetum degeneration, anther development and pollen exine formation. The product generated by OsSTRL2 is involved in the maturation of anther wax crystals, PCD of tapetal cells, and formation of pollen exine.

The mutation in *Ms45* gene, a maize homolog of *OsSTRL2* (Fig. 2), causes the absence of pollen. Immunological analyses showed that MS45 was localized to the tapetal cells during microsporogenesis^35^. Our results indicated that *OsSTRL2* was specifically expressed in tapetum and microspores via GUS reporter analysis and RNA *in situ* hybridization (Fig. 6), suggesting that OsSTRL2 and MS45 might share this tapetal localization and play the conserved roles during exine biosynthesis. PKSA, PKSB, and TKPR1 are required for sporopollenin biosynthesis in *Arabidopsis* ^51, 52, 55^. These enzymes are immunolocalized to the ER of anther tapetal cells^56^. Similarly, our results also showed that OsSTRL2 was mainly localized in the ER (Fig. 7), whereas the STR1 orthologue in periwinkle was simultaneously targeted to the chloroplast, vacuole and ER in transgenic tobacco^57^. These results suggested that the ER of tapetum might be an important site for the proteins regulating pollen wall formation, and therefore OsSTRL2 might participate in this essential network by influencing the tapetum degeneration, anther wax crystal development and pollen exine formation (Fig. 8).

The structural characterizations of STR1 deciphered the 3D structure of the active site of strictosidine synthase and the details of its reaction mechanism^3, 4, 7^. The structure of STR1 contains six-bladed β-propellers and three α-helices. The substrate-binding pocket of STR1, in which several vital residues (Fig. 2B) are involved, links the active center to the surface of the substrate molecule. In addition, the structural analysis and site-directed mutagenesis experiments found that Glu-309 (Fig. 2B) in the residues of STR1 binding pocket is a key catalytic residue^4, 7^. Among the typical STR family, these motifs and residues are important and conserved because they provide the basic structure of the protein^7^. However, OsSTRL2 lacks Glu-309, which is the key catalytic residue of typical strictosidine synthase STR1. OsSTRL2 also exhibits differences in some residues in the substrate-binding pocket (Fig. 2B), although it contains the motifs for β-propeller folds and the residues Cys-89 and Cys-101, which form a disulfide bridge and pull two out of three α-helices together (Fig. 2B). This finding suggested the possibility that OsSTRL2 might have no or have low catalytic activity as presented in the typical strictosidine synthase. Similar to OsSTRL2, AtSsl7 and AtSsl14 are *Arabidopsis* strictosidine synthase-like proteins that lack the Glu-309 and some other residues in the substrate-binding region (Supplementary Figure 4) and do not have strictosidine synthase activity^33^. This result might offer experimental evidence to our hypothesis on the catalytic activity of OsSTRL2.

*LAP3* is the homologous gene of *OsSTRL2* in *Arabidopsis* (Fig. 2) and is one of the *ATSsl* genes^4^. LAP3 has a relatively low similarity to the protein sequence of STR1^34^. Our results also revealed a comparatively low amino acid sequence identity between OsSTRL2 and STR1 (Fig. 2). These findings indicated that both LAP3 and OsSTRL2 have evolutionary divergence with STR1. In addition, Kibble *et al*. found that all members of the ATSsl family lack the Glu-309 and suggested that this family might not have strictosidine synthase enzyme activity^4^. The absence of this critical catalytic residue also occurs in all OsSTRL family members of rice (Supplementary Figure 1). These differences might bring variations in the substrate specificity and/or enzymatic activity for OsSTRL family members, as well as the possibilities that those different members might participate in other biochemical pathways in plants^11^. Although OsSTRL2 lacks some the key residues of STR, we propose that OsSTRL2 is an atypical strictosidine synthase because it processes the basic framework of a STR protein (e.g., β-propeller folds and α-helices).

In summary, this study identified 21 rice *OsSTRL* genes through genome-wide analysis. The 21 *OsSTRL* members were clustered into two major groups and four clades in group I. The expression pattern analysis showed that only *OsSTRL2* was specifically expressed in the developing anther. *OsSTRL2* loss-function in rice caused the detention of tapetum degeneration, abnormal wax crystals, and orbicules of anther and the absence of pollen exine tectum, which led to the knockout mutant male sterility. Based on the accumulation of *OsSTRL2* in tapetal cells and microspores at the transcript level and the ER localization of OsSTRL2-YFP protein, OsSTRL2 might participate in the regulation network of pollen wall formation that occurs in the ER of tapetum. By combining the results of protein sequence alignment, we consider OsSTRL2 as a rice atypical strictosidine synthase that play key roles in anther development and pollen wall formation. Our work also provides a new perception into mechanisms of anther and pollen developmental processes and enhances our understanding of the regulation of pollen exine formation.

## Materials and Methods

### Plant materials and growth conditions

All rice (*Oryza sativa*) plants used in this study were naturally grown and maintained in the experimental field of the Rice Research Institute, Sichuan Agricultural University, Wenjiang, China.

### Genome-wide sequence analysis of rice STR-like genes and phylogenetic analysis

Using the full-length STR1 protein sequence in *Rauvolfia Serpentine* as a query, a BLASTP search of the japonica-type rice genome (RGAP; http://rice.plantbiology.msu.edu/)^28^ was performed to forecast the 21 OsSTRL candidates. The amino acid sequences of these candidates were subsequently used for the SMART search^29, 58^ and were aligned by using Clustal W software (www.ebi.ac.uk/Clustalw) to further confirm the accuracy. The OsSTRL2-related protein sequences in different species were identified by Phytozome Blast (https://phytozome.jgi.doe.gov) with default parameters using the full-length amino acid of OsSTRL2 as a query, and the sequences aligned with Clustalw2^31^. In addition to the 1,000 bootstrap replications, the MEGA5 program used the NJ method with default parameters to generate all the phylogenetic trees^32^.

### CRISPR/Cas9-mediated mutation and phenotype association assay

To verify the *OsSTRL2* function, we generated a gRNA construction, wherein the gRNA and the plant-optimized Cas9 were driven by the rice U3 and maize UBI promoter, respectively^59^. Plasmid was introduced into the WT (*Nipponbare*). The DNA isolated from the leaves of transgenic plants was subjected to PCR and sequencing analysis with the primer set OsSTRL2-gRNA-seq (see Supplementary Table S3) to evaluate whether the mutation occurred. To verify the association between the candidate mutation site in *OsSTRL2* and the male sterile phenotype, we observed the target site sequence of all the CRISPR/Cas9 transgenic plants via direct or cloned sequencing of the PCR products, which were amplified using the primer set OsSTRL2-gRNA-seq (statistical results are shown in Supplementary Table 1). The co-segregation results were further confirmed in several F2 populations that were generated by backcrossing these mutants with the WT.

### Phenotypic characterization

Transverse sections of the anther development analysis were performed as described previously^19^. For the SEM analysis of anther and pollen, the samples at stage 12 were prepared as described by Qin *et al*.^25^ and then examined with a JSM-7500F scanning electron microscope.

### Expression analysis

Digital expression pattern analysis of 21 *OsSTRL* members was performed by using the public RNAseq data from NGSTD (http://rice.plantbiology.msu.edu/expression.shtml) to draw a heat map with the OmicShare tools, a free online platform for data analysis (http://www.omicshare.com/tools/). Total RNAs from a variety of rice tissues were extracted using the TriPure isolation reagent (Roche, Indianapolis, USA). cDNAs were reverse-transcribed using the Transcriptor First-Strand cDNA Synthesis Kit (Roche, Indianapolis, USA). Semi-RT-PCR was performed with the program involving pre-denaturation at 95 °C for 2 min, 29 or 30 cycles of the reaction at 95 °C for 30 s, 57 °C for 30 s and 72 °C for 30 s. Another step of at 72 °C for 7 min was conducted for the final extension, and the PCR amplification results were run on 1% agarose gel. The gel image was obtained by using Molecular Image Gel Doc XR + image analysis system (Bio-Rad, Hercules, CA). qPCR experiments conducted by using a Bio-Rad CFX96 real-time PCR System (California, USA) as described by Li *et al*.^60^, the rice actin gene was used as the internal control, and the fold change for gene expression was calculated as described previously^61^. All primers used in this study are listed in Supplementary Table 3.

### Promoter-GUS reporter assays and RNA ***in situ*** hybridization

For the transcript level tissue localization of *OsSTRL2*, a construct of OsSTRL2pro::GUS was generated through pCAMBIA1300 wherein the native OsSTRL2 promoter drove the GUS gene. The OsSTRL2pro::GUS construct was introduced into the *Agrobacterium tumefaciens* strain EHA105 and then transferred into the WT. Histochemical GUS staining was performed as described previously^62^. A 406 bp fragment of *OsSTRL2* cDNA was amplified using specific primers (Supplementary Table 3) and cloned into pBluescript-SK vector (Stratagene) to generate the OsSTRL2-specific antisense probe. The fragment was transcribed with T7 RNA polymerase. *In situ* hybridizations including tissue embedding, hybridization and signal detection were conducted as described by Chen *et al*.^63^.

### Subcellular localization of OsSTRL2

The full-length cDNA of *OsSTRL2* was cloned into pA7-YFP vector to generate a 2 × 35S::OsSTRL2-YFP cassette which fused the C-terminal of OsSTRL2 with YFP under the control of the cauliflower mosaic virus double 35 S promoter. The entire 2 × 35 S::OsSTRL2-YFP cassette was then inserted into the pCAMBIA1300. The RFP with the C-terminal extension-KDEL served as the ER marker under the control of the constitutive cauliflower mosaic virus double 35 Spromoter^64^. These plasmids were individually expressed or co-expressed in tobacco leaf epidermis cells by agrobacterium-mediated infiltration. YFP and RFP signals were visualized with a confocal scanning microscope (Nikon A1, Kanagawa, Japan) 72 h after infiltration.

## Electronic supplementary material

Supplementary Information
