# Supplementary material for: An atypical strictosidine synthase, OsSTRL2, plays key roles in anther development and pollen wall formation in rice
Source: Sci Rep. 2017 Jul 31;7:6863. doi: 10.1038/s41598-017-07064-4 (PMC5537339; doi:10.1038/s41598-017-07064-4)
Supplement: Supplementary file 1 — Supplementary Information [file 41598_2017_7064_MOESM1_ESM.pdf]

Title:

An atypical strictosidine synthase, OsSTRL2, plays key roles in anther development and pollen wall formation in rice

Authors:

Ting Zou[1,2#] Shuangcheng Li[1,2,3#*] Mingxing Liu[1,2] Tao Wang[1,2] Qiao Li[1,2] Qiao Xiao[1,2] Dan Chen[1,2] Yanling Liang[1,2] Jun Zhu[1,2,3] Yueyang Liang[1,2] Qiming Deng[1,2,3] Shiquan Wang[1,2,3] Aiping Zheng[1,2] Lingxia Wang[1,2,3] Ping Li[1,2,3*]

[1] State Key Laboratory of Hybrid Rice, Sichuan Agricultural University, Chengdu 611130, China;

[2] Rice Research Institute, Sichuan Agricultural University, 211 Huimin Road, Wenjiang 611130, Sichuan, China;

[3] Key Laboratory of Crop Genetic Resources and Improvement, Sichuan Agricultural University, Ministry of Education, Ya'an 625014, Sichuan, China.

[#] These two authors contribute equally to this work.

[*] Corresponding author: Shuangcheng Li, E-mail: lisc926105@163.com and Ping Li E-mail: liping6575@163.com

SUPPLEMENTARY TABLES 1-4

Supplementary Table 1. Phenotype and genotype association analyses in CRISPR/Cas9 transgenic plants.

	Homo-WT	Heterozygous	Biallelic	Homo-MT	Total
Normal fertility	7	3	0	0	10
Male sterility	0	0	15	4	19

The numbers in this table indicate the numbers of different genotype transgenic plants in different phenotypes.

Supplementary Table 2. The detailed information of 4 different mutation types in Fig.3

Mutation type	Sequence (- strain, 5' to 3')	Numbers of plants	Genotype of plants	Protein sequence
WT sequence	GCTCCTCACCGACCCGTTCC	10	Homo-WT or heterozygous	WT

2bp insertion	GCTAACCTCACCGACCCGTTCC	6	Bialleic or homo-MT	Premature
1bp insertion	GCTACCTCACCGACCCGTTCC	6	Bialleic or Homo-MT	Premature
2bp deletion	GCT--TCACCGACCCGTTCC	9	Heterozygous or bialleic or homo-MT	Premature
1bp deletion	GCT-CTCACCGACCCGTTCC	11	Heterozygous or bialleic or homo-MT	Premature

The numbers in this table indicate the numbers of transgenic plants in different mutation types. The red characters or dashes indicate the insertion or deletion events generated by CRISPR-Cas9 in mutants.

Supplementary Table 3. All primers used in this study.

Primer ID	Sequence(5' to 3')
OsSTRL2-RT-F	TGGCTGCGAACGCTCTACT
OsSTRL2-RT-R	CTCTGCTCTTCCAACGGGTAA
OsSTRL2-QPCR-F	CGACACGAGCACGAGATA
OsSTRL2-QPCR-R	ACGCCATTCGGGAAGACCA
OsACTIN-RT-F	GGAACTGGTATGGTCAAGGC
OsACTIN-RT-R	AGTCTCATGGATACCCGCAG
OsACTIN-QPCR-F	GCTATGTACGTCGCCATCCA
OsACTIN-QPCR-R	GGACAGTGTGGCTGACACCAT
OsSTRL2-promoter-F	gagatctacagcgctaagcttAAGGTTTAGGGCTTGTTCAC
OsSTRL2-promoter-R	ggactgaccacccggggatccGGAGATCAGGAGCGAGGGA
OsSTRL2-YFP-F	accagtctctctctcaagcttATGGAAGAGAAGAAGCAGCAGC
OsSTRL2-YFP-R	gctcaccatactagtggatccATCACCAAGCACGTTGCTGC
OsSTRL2-gRNA-seq-F	TCTTCCCTCGCTCCTGAT
OsSTRL2-gRNA-seq-R	TGGCACAAACTTTCTCCG
OsSTRL2-CRISPR-F	ggcaGGAACGGGTCGGTGAGGAGC
OsSTRL2-CRISPR-R	aaacGCTCCTCACCGACCCGTTCC
OsSTRL2-Antisense-F	cgggatccATGCGGTACTGGCTGGAAGG
OsSTRL2-Sense-R	ggaattcCCAACGGGTAAGGGATCGTG
OsSTRL1-RT-F	AGCAGATCAAGACCACCGACAC
OsSTRL1-RT-R	TGAAGTAGGCATCACCCGTAGC
OsSTRL3-RT-F	TCACTGACAGCAGCATCCACT
OsSTRL3-RT-R	CTGACATTGCGACTGACCATT
OsSTRL4-RT-F	GAGGTGGTGACGACGGAGAC
OsSTRL4-RT-R	TGCGGGTGGCATAGCAGAG
OsSTRL5-RT-F	ATGGGATTGATGCGAGTTG
OsSTRL5-RT-R	TTCGGGTAGGTTATGTTGGA
OsSTRL6-RT-F	TACATCGCCGACGCCTAC

OsSTRL6-RT-R	TGAGCCTTGAATCCAATACCT
OsSTRL7-RT-F	ATGGGATTGATGCGAGTTG
OsSTRL7-RT-R	TTCGGGTAGGTTATGTTGGA
OsSTRL8-RT-F	CCGCCTGATGAAGTACGACC
OsSTRL8-RT-R	AATTCCACCGAACCCAAATAAA
OsSTRL9-RT-F	GTGCCGTTCAAGTTCACCAA
OsSTRL9-RT-R	CACCCAGTAGCCTCCCTTCC
OsSTRL10-RT-F	GTGCCGTTCAAGTTCACCAA
OsSTRL10-RT-R	CACCCAGTAGCCTCCCTTCC
OsSTRL11-RT-F	GTGCCGTTCAAGTTCACCAA
OsSTRL11-RT-R	CACCCAGTAGCCTCCCTTCC
OsSTRL12-RT-F	CTTCTTCGAGTACCGCTTCACC
OsSTRL12-RT-R	CACATTCCCTTCAGAGTCACCA
OsSTRL13-RT-F	GCTCGCTTCCCTCCCAACA
OsSTRL13-RT-R	CGCGTCCATTCTCATCCACTTT
OsSTRL14-RT-F	TCACCGACAGCAGCACCA
OsSTRL14-RT-R	GCTCAGCGTCACAGCCCTA
OsSTRL15-RT-F	CTTCACCGATAGCAGCACCACG
OsSTRL15-RT-R	CGCCTCCACCCTTCTTCTTCC
OsSTRL16-RT-F	TGGACCGAGTTTGCCTACAAC
OsSTRL16-RT-R	TTTCACCCTCCTCCGATACG
OsSTRL17-RT-F	ACAGGGCGGCTGCTCTGGTA
OsSTRL17-RT-R	GACCCAAAGCGTGCTGTTCC
OsSTRL18-RT-F	TGGACCGAGTTCGCCCACA
OsSTRL18-RT-R	AGCAGGTAGTCGCTCCGCTTG
OsSTRL19-RT-F	TCCCTGACAACATCCGCTACG
OsSTRL19-RT-R	GATCCTGCTGAGGTACGGTTTG
OsSTRL20-RT-F	GACAGGAAGCCCATTCAG
OsSTRL20-RT-R	GATGCTCATAGCCAAACC
OsSTRL21-RT-F	GCGTTGTTGGTGTCCCTG
OsSTRL21-RT-R	GCTCCCGTTTCGTGTCCT

The construction adapter is in lower character.

Supplementary Table 4. The information of motifs shown in Supplementary Figure 2B.

Motif	Width	Best possible match
1	41	CGRPLGLQFHNKTGNLYIADAYMGLMRVGPRGGEATVLATE
2	29	VPFNFTNGVDIDQVTGDVYFTDSSTTYQR
3	41	TGRLMKYDPRTNQVTVLQSGMTYPNGVAMSADRSHLVVCHT
4	26	YWIKGPKAGKSEPFAELPGYPDNVRP
5	50	GYWVALHREKYETPYGPDTHLLAMRIGRKGKILQQMRGPKNVRPTEVIER
6	17	DGPYTGVSDGRIMRWNG
7	37	MVVEELTGFKFKTISEVEEQNGKLWIGSVDTPYIGLY
8	21	RSQHLPLPGPVTGPESVAFDG
9	15	GWTTFAYNPNYWKIK
10	11	SQHEQVTATKD

SUPPLEMENTARY FIGURES 1-4

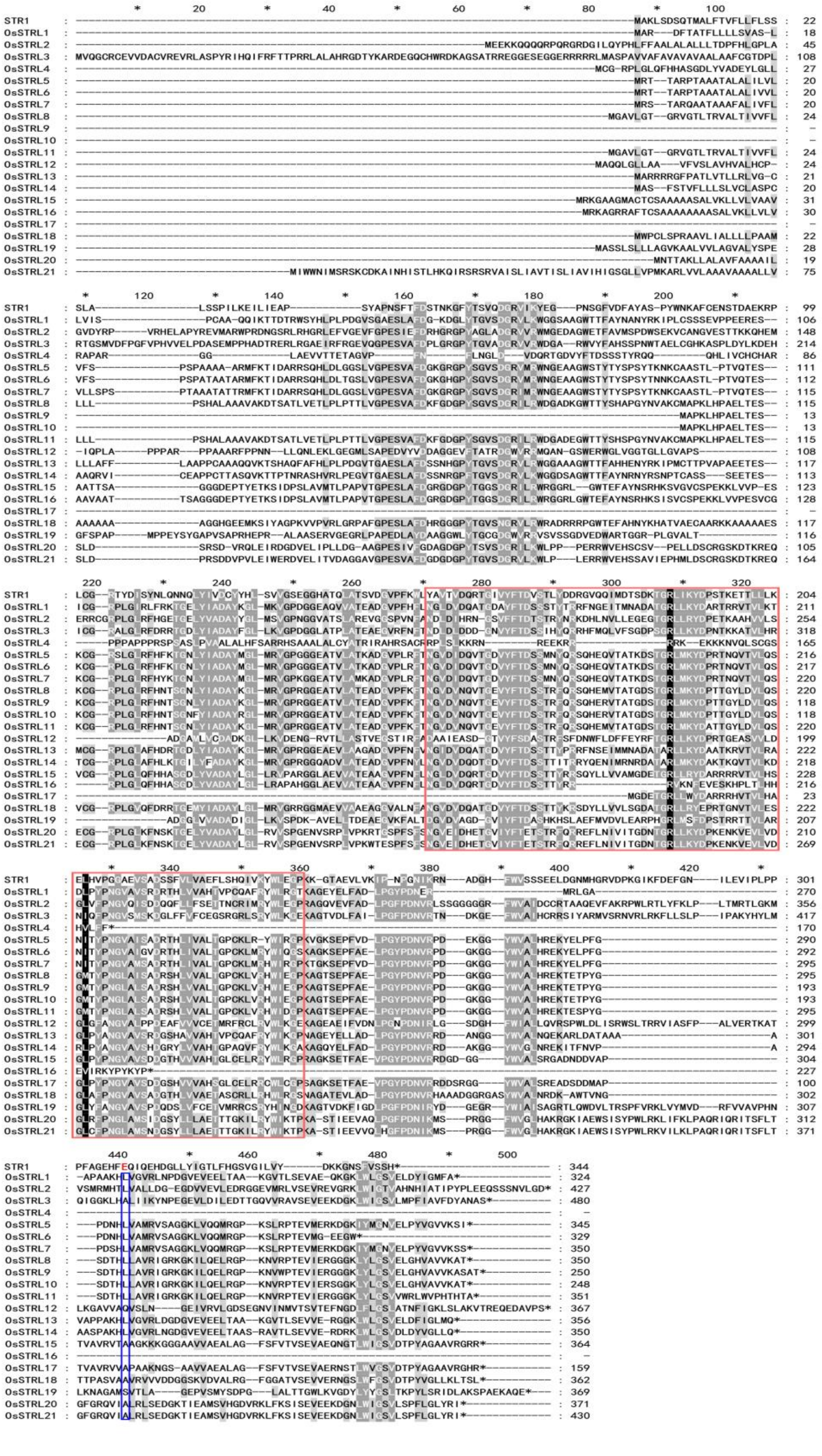

Supplementary Figure 1. Sequence alignment of 21 OsSTRL proteins in rice. Red

frames outlines the conserved strictosidine synthase domain. The sequences were displayed using BOXSHADE. Red character indicates the key catalytic residue (Glu-309) of typical STR. Blue frame outlines the variation of residues at catalytic residue position of OsSTRLs.

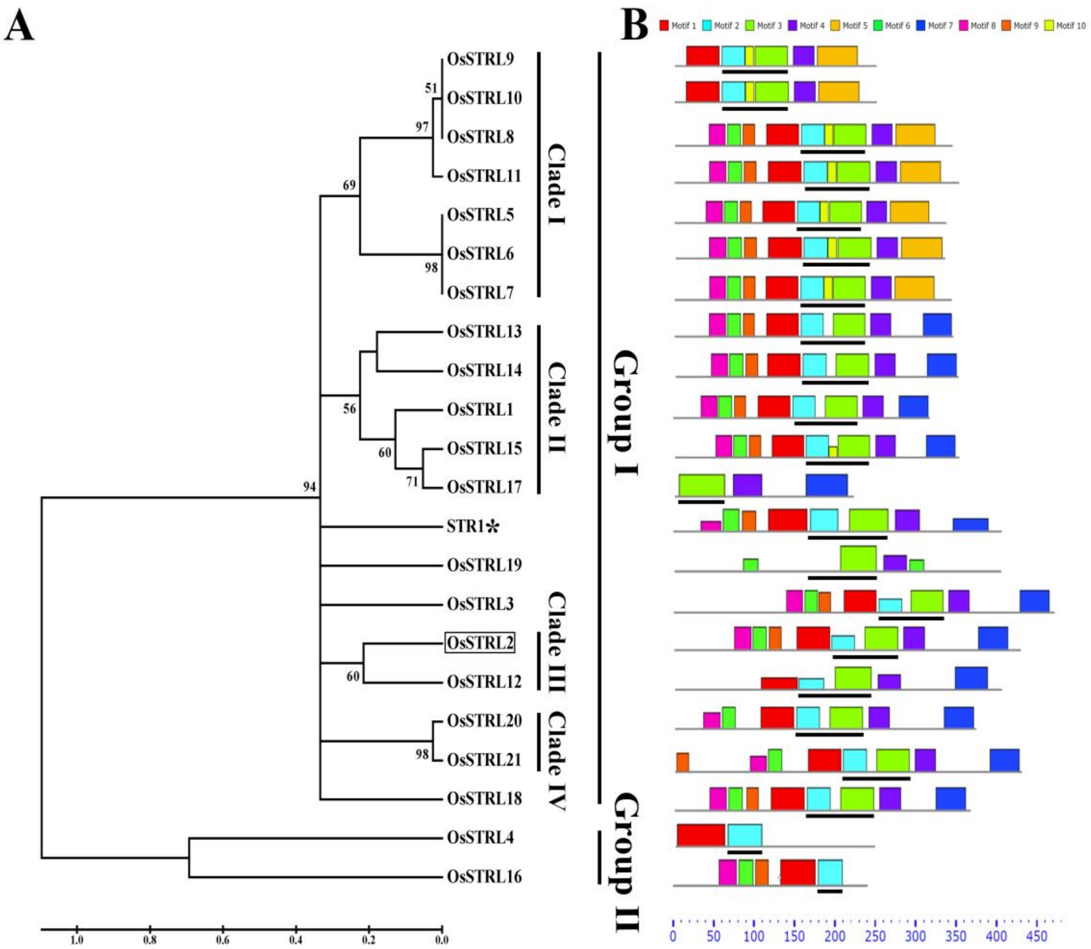

Supplementary Figure 2. Protein phylogeny and domain analysis of 21 rice OsSTRL members and STR1. (**A**) Phylogenetic analysis of 21 rice OsSTRL members and STR1 was performed by using MEGA5 with the neighbor-joining method based on the alignment results in Supplementary Fig. S1. Bootstrap values are the percentage of 1,000 replicates. The 21 rice OsSTRL protein sequences are manually grouped into two major groups and four clades in group I. (**B**) Distribution of motifs in 21 rice OsSTRL members and STR1 identified by employing MEME motif search tool. Each motif is represented by a colored box. Black lines under motifs indicate locations of the strictosidine synthase domain. The information of motifs were listed in Supplementary Table 4.

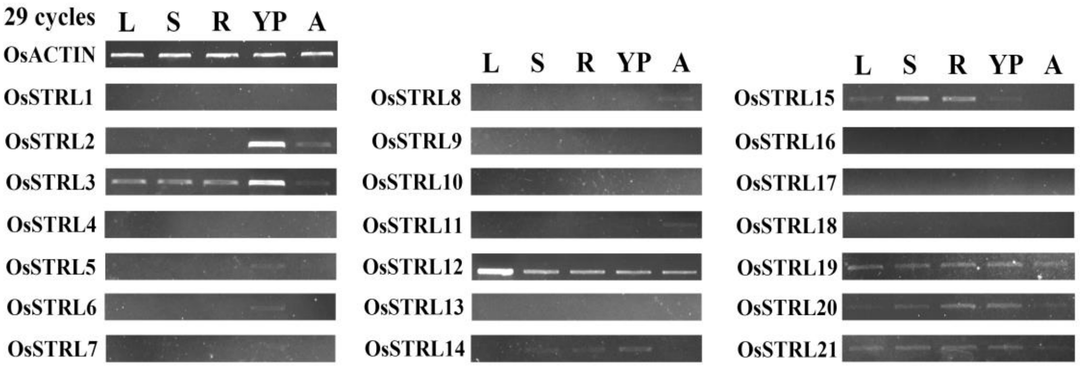

Supplementary Figure 3. Expression analysis of *OsSTRL* genes in different tissues using Semi RT-PCR. Semi RT-PCR was performed using primer specific for the *OsSTRL* genes with 28 PCR cycles. PCR products were run on 1 % agarose gels. *OsACTIN* were used as the internal standard for each gene. Sources of the samples are as follows: leaf (L), stem (S), root (R), young panicles (YP), and anther (A). The primers used for expression analysis of *OsSTRL* genes are listed in Supplementary Table 3.

```
ATSsl7     ----------------------------------KLDTF----------DPAIVPSDAFT
OsSTRL2    MEEKKQQQQRPQRGRDGILQYPHLFFAALALALLLTDPFHLGPLAGVDYRPVRHELAPYR
STR1       MAKL---------SDSQTMALFTVFLLFLSSSLAL-------------------------
AtSsl14    ------------------------------------------------------------

ATSsl7     SSATSLPPLINDEFLTGAEFIGVGLLNIPEDIAYHKESNLIYTGCVDG-----------W
OsSTRL2    EVMARWPR-DNGSRLRHGRLEFVGEVFGPESIEFDRHGRGPYAGLADGRVVRWMGEDAGW
STR1       ------------SSPILKEILIEAPSYAPNSFTFDSTNKGFYTSVQDGRVIKYEGPNSGF
AtSsl14    ------------DDASFQKLPVPDKRSGPESFAFDST-GGFYTGVSGGKILKYV-PGKGY

ATSsl7     VKRVKVADSVNDSVVE-----------DWVNTGGRPLGIAFG-IHGEVIVADVHKGLLNI
OsSTRL2    ETFAVMSPDWSEKVCANGVESTTKKQHEMERRCGRPLGLRFHGETGELYVADAYYGLMSV
STR1       VDFAYASPYWNKAFCENSTDA------EKRPLCGRTYDISYNLQNNQLYIVDCYYHLSVV
AtSsl14    VDFAQITDSSNSAWCNGALGT------AFAGKCGRPAGIALNSKTGDLYVADAPLGLHVI

ATSsl7     SGDGKKTELLTDEADGVKFKLTDAVTVA-DNGVLYFTDASYKYTLNQLSLDMLEGKPFGR
OsSTRL2    GPNGGVATSLAREVGGSPVNFANDLDIH-RNGSVFFTDTSTRYNRKDHLNVLLEGEGTGR
STR1       GSEGGHATQLATSVDGVPFKWLYAVTVDQRTGIVYFTDVSTLYDDRGVQQIMDTSDKTGR
AtSsl14    SPAGGLATKLADSVDGKPFKFLDGLDVDPTTGVVYFTSFSSKFGPREVLIAVGLKDASGK

ATSsl7     LLSFDPTTRVTKVLLKDLYFANGITISPDQTHLIFCETPMKRCSKYYISEERV---EVFT
OsSTRL2    LLRYDPETKAAHVVLSGLVFPNGVQISDDQQFLLFSETTNCRIMRYWLEGPRAGQVEVF-
STR1       LIKYDPSTKETTLLLKELHVPGGAEVSADSSFVLVAEFLSHQIVKYWLEGPKKGTAEVL-
AtSsl14    LFKYDPATKAVTELMEGLSGAAGCAVSSDGSFVLVSEFIKSNIKKYWIKGPKAGTIEDF-

ATSsl7     QSLPGYPDNIRY---DGDGHYWIALPSGVTTLWNISLKYPFLRKLTAMVA----KYGVDL
OsSTRL2    ADLPGFPDNVRLSSGGGGGRFWVAIDCCRTAAQEVFAKRPWLRTLYFKLPLTMRTLGKMV
STR1       VK-IPNPGNIKR---NADGHFWVSSSEELD---------------------------GNM
AtSsl14    SSLVSNPDNIRRV--GSTGNFWVASVVN------------------------------KV

ATSsl7     MFMENAGVLQVDLDGNPIAYYHD---PKLSHIATCDKIGKYLYCGSLSQSHILRLDLLKY
OsSTRL2    SMRMHTLVALLDGEGDVVEVLEDRGGEVMRLVSEVREVGRKLWIGTVAHNHIAT---IPY
STR1       HGRVDPKGIKFDEFGNILEVIPLPPPFAGEHFEQIQEHDGLLYIGTLFHGSVGI---LVY
AtSsl14    VMPTDPRAVKLDANGKVLQTIFLKNEFGNTLLSEVNEFNGHLYIGTLTGPFAGV---MKL

ATSsl7     PAQNKKL*------
OsSTRL2    PLEEQSSSNVLGD*
STR1       DKKGNSFVSSH*--
AtSsl14    *-------------
```

Supplementary Figure 4. The amino acids alignment of ATSsl7, ATSsl14, OsSTRL2 and STR1. Red background characters indicate the motifs for β-propeller folds. Light blue background characters indicate the conserved residues forming Disulfide Bridge that pulls two α-helices together. Green background characters indicate the residues contact with the terpenoid part of secologanin. Yellow background characters indicate the residues contact with indole part of strictosidine. Deep blue background characters indicate the residues contact with the glucose moiety. Pink background character indicates the key catalytic residue of typical strictosidine synthase.
